# Optimizing Shade Cultivation Method and Irrigation Amount to Improve Photosynthetic Characteristics, Bean Yield, and Quality of Coffee in a Subtropical Monsoon Climate

**DOI:** 10.3389/fpls.2022.848524

**Published:** 2022-04-29

**Authors:** Kun Hao, Xiaogang Liu, Xiukang Wang, Liangjun Fei, Lihua Liu, Feilong Jie, Yilin Li, Qiliang Yang, Yunhui Shan

**Affiliations:** ^1^Faculty of Modern Agricultural Engineering, Kunming University of Science and Technology, Kunming, China; ^2^College of Life Science, Yan’an University, Yan’an, China; ^3^State Key Laboratory of Eco-Hydraulic in Northwest Arid Region, Xi’an University of Technology, Xi’an, China; ^4^Dehong HeiRou Coffee Co., Ltd., Dehong, China

**Keywords:** coffee, shade cultivation, deficit irrigation, yield, quality

## Abstract

Reasonable water and light management technology can improve economic benefits, coffee yield, and quality. We used cluster analysis and principal component analysis to evaluate and optimize the water and light management technology with high coffee yield, quality, and economic benefits in a subtropical monsoon climate region of China. The experiment was arranged in a randomized complete block design with two factors (3 irrigation levels × 4 shade cultivation treatments) replicated four times during 2016–2017. The irrigation levels consisted of full irrigation (FI) and two deficit irrigations (DI_*L*_: 75% FI, DI_*S*_: 50% FI). The shade cultivation treatments consisted of no shade cultivation (S_0_) and three shade cultivation modes (S_*L*_: intercropping with four lines of coffee and one line of banana; S_*M*_: intercropping with three lines of coffee and one line of banana; S_*S*_: intercropping with two lines of coffee and one line of banana). The results showed that the effects of irrigation level and shade cultivation mode on growth, crop yield, most of the photosynthetic characteristics, and nutritional quality were significant (*p* < 0.05). Regression analysis showed that the leaf radiation use efficiency (*RUE*) showed a significant negative exponential relation or logistic-curve variation with photosynthetically active radiation (*PAR*). The bean yield increased with an increase of the shade degree when water was seriously deficient, whereas it first increased and then decreased with an increase of the shade degree under FI and DI_*L*_. Based on both cluster analysis and principal component analysis, the FIS_*S*_ treatment resulted in the highest comprehensive quality of coffee, followed by the FIS_*M*_ treatment; the DI_*S*_S_0_ treatment obtained the lowest quality. Compared with the FIS_0_ treatment, the FIS_*M*_ treatment increased the 2-year average bean yield and net income by 15.0 and 28.5%, respectively, whereas the FIS_*S*_ treatment decreased these by 17.8 and 8.7%, respectively. To summarize, FIS_*S*_ treatment significantly improved the nutritional quality of coffee, and FIS_*M*_ treatment significantly increased the dry bean yield and economic benefits of coffee. The results of the study could provide a theoretical basis for water-saving irrigation and shade cultivation management of coffee in a subtropical monsoon climate region of China.

## Introduction

Coffee is one of the most popular hot drinks in the world, with three times as much coffee consumed as cocoa and four times as much as tea (Ruesgas-Ramón et al., 2020). In 2016, the coffee yield was 1.60 × 10^8^ kg and the export of beans was 8.27 × 10^7^ kg (export value of USD 9.04 × 10^8^) in China (Liu et al., 2018). China had become one of the largest coffee-producing areas worldwide (Rigal et al., 2020). The coffee yield of Yunnan was 1.58 × 10^8^ kg, accounting for 98.8% of the yield in China. Arabica coffee is the most common coffee plant in the Yunnan province. Coffee production has been the main economic source for local farmers. Only by improving the yield and quality of coffee can farmers’ economic income be guaranteed to improve their quality of life. The subtropical monsoon climate region of China has abundant light and heat resources, strong seasonal drought, strong light, and long average sunshine duration (Zhang et al., 2017). Presently, coffee production is mainly dependent on rain or flood irrigation under natural light, hence, the disconnect between the available water and light tends to restrict the efficient production of coffee in the subtropical monsoon climate region of China.

Deficit irrigation (DI) is a new water-saving irrigation technique based on various physiological regulation theories (Zhou et al., 2017), aiming to optimize irrigation by preserving irrigation water, labor, and energy inputs, maximizing water-use efficiency (Minhas et al., 2020). DI saves a considerable amount of irrigation water, and moderate DI can maintain or increase crop yield while improving quality (Al-Ghobari and Dewidar, 2018; Yu et al., 2020). Studies have demonstrated that the effects of DI on the vegetative growth of coffee trees, total sugar, protein, fat, crude fiber, caffeine, and chlorogenic acid content of green coffee beans were significant, but there was no significant difference between light deficit irrigation and full irrigation (Liu et al., 2016). Other studies have shown that increasing the amount of irrigation can significantly increase coffee yield (Byrareddy et al., 2020); partial root-zone irrigation can greatly improve water-use efficiency and coffee bean quality (Minhas et al., 2020); and moderate DI can improve the protein, fat, and chlorogenic acid content of green coffee beans (Liu et al., 2016). Moreover, Perdoná and Soratto (2015a) reported that irrigated coffee-macadamia intercropping is the most financially superior treatment.

Intercropping coffee plants with shade trees is regarded as a climate-smart agricultural practice (Ortiz-Gonzalo et al., 2018); reasonable shade cultivation improves the regulation of light, heat, water, soil, fertilizer, and other factors affecting crop growth, hence, providing a suitable growth environment for coffee (Gruda, 2019) and preventing premature senescence and falling of leaves due to excessive oxidation (Bartoli et al., 2013; Shi et al., 2022). Additionally, reasonable shade cultivation delays the maturation of coffee berries resulting in superior bean filling and larger beans and increasing the yield and coffee quality (Pinard et al., 2014; Mariño et al., 2016). Furthermore, shade trees can help to diversify farmers’ income and thereby increase food security (Anderzén et al., 2020; Marcheafave et al., 2020). Farmers have intercropped corn, beans (Huang et al., 2020), banana (van Asten et al., 2011), cordia (Rahn et al., 2018), macadamia (Perdoná and Soratto, 2016), Tabebuia rosea, and Simarouba glauca (Sarmiento-Soler et al., 2019) with coffee to achieve coffee shade cultivation. The purpose of this study was to intercrop banana plants in coffee plantations, with the photophilic bananas located in the upper layer and shade-tolerant coffee located in the lower layer, so that sunlight could be utilized at multiple levels. However, in the subtropical monsoon climate region of China, numerous topics, including the actual productivity, quality, and comprehensive economic benefit of coffee sheltered under bananas that provide various levels of shade, remain to be investigated.

Irrigation can promote coffee growth, the highest yield and economic benefits were achieved when coffee was intercropped with macadamia (Perdoná and Soratto, 2015b). Liu et al. (2018) demonstrated that water-use efficiency and the nutritional quality of coffee beans were improved and the yield of coffee was not reduced when 75% of the full irrigation amount was used under 70% of natural light intensity. However, how DI can be combined with banana shade cultivation to obtain stable yield and improve the quality and comprehensive benefits of coffee in a subtropical monsoon climate region remains unclear and deserves further study. The study hypothesizes that the appropriate combination of DI and shade cultivation could improve the photosynthesis, yield, quality, and economic benefits of coffee. The effects of DI on photosynthetic characteristics, bean yield, nutritional quality, and economic benefit of coffee were investigated under different banana shade cultivation modes, and the nutritional quality of the coffee was comprehensively evaluated using principal component analysis and cluster analysis to determine the optimal interplanting mode and irrigation level. The results could provide a scientific basis for the agricultural water supply and shade cultivation management of coffee in the subtropical monsoon climate region of China.

## Materials and Methods

### Experimental Site and Materials

**FIGURE 1:**
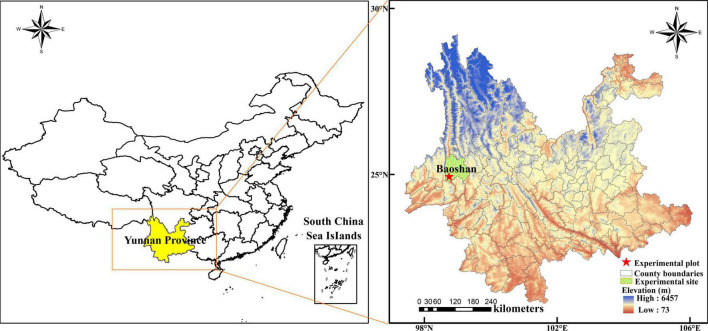
The location of the experimental site.

Field experiments were conducted from March 2016 to January 2018 in Baoshan, Yunnan, southwest China (latitude 25°4′ N, longitude 99°11′ E, altitude 799 m a.s.l.). Rainfall during the experimental period was 1,394.9 mm. Among them, the rainfall was 576.3 mm from 1 March 2016, to 31 January 2017, and 818.6 mm from 1 February 2017, to 31 January 2018. The daily mean maximum and minimum temperatures were 32.3 and 10.4°C, respectively. The location of the experimental site is given in Figure 1.

Five-year-old coffee (*C. arabica* L. cv. Caturra) plants with similar growth were selected as the experimental material. The plant height was 171–179 cm, stem diameter was 22.27–24.34 mm, plant spacing was 1.5 m, and row spacing was 2.0 m (3,333 plants ha^–1^). Medium-sized banana plants (Williams 8818) that are fast-growing and easy to control, provide considerable canopy shade, and exhibiting strong coffee symbiosis were selected as the shade plant species. Banana seedlings with similar growth were planted in the experimental area on 9 March 2016; the plant height was 50–55 cm, and the leaf number was five or six.

The soil in the experimental field was a reddish-brown sandy loam soil that had a soil bulk density of 1.40 g cm^–3^, a field capacity of 23%, a pH of 6.7, an organic matter content of 20.2 g kg^–1^, an available N content of 106 mg kg^–1^, an available P content of 12.6 mg kg^–1^, and an available K content of 56 mg kg^–1^ in the topsoil (0–40 cm).

### Experimental Design and Method

**FIGURE 2:**
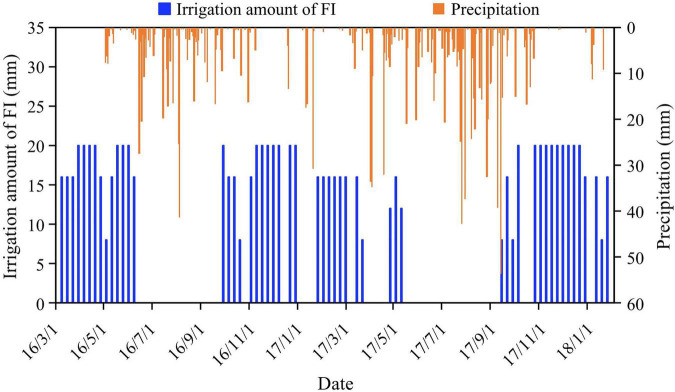
Precipitation and irrigation amount of full irrigation (FI) in the experimental periods.

With full irrigation (FI) under natural light intensity as control, three irrigation levels and four shade cultivation modes were employed in the field experiments. A complete combination design was used with 12 treatments (i.e., 3 × 4), and each treatment was applied in 4 plots, with 48 plots in total. The three irrigation levels were FI, light deficit irrigation (DI_*L*_: 75% FI), and severe deficit irrigation (DI_*S*_: 50% FI). The irrigation level FI was determined from the monthly water demand data of coffee (Chen et al., 1995) and precipitation, and FI was equal to the coffee water consumption minus effective rainfall. Irrigation frequency was performed every 7 days, but the irrigation date was postponed when a rainfall event occurred. Surface drip irrigation was used under a system working pressure of 0.1 MPa, and pressure compensating emitters with a flow of 2 L h^–1^ were installed on both sides of the capillary tube of each coffee tree (with the tube located 0.4 m from the base of the tree). During the experiment, the FI, DI_*L*_, and DI_*S*_ irrigation amounts were 492.0, 369.0, and 246.0 mm, respectively, from 1 March, 2016, to 31 January 2017, and 453.0, 339.8, and 226.5 mm, respectively, from 1 February 2017, to 31 January 2018. Figure 2 illustrates the precipitation and irrigation amounts of FI in the experimental periods.

**FIGURE 3:**
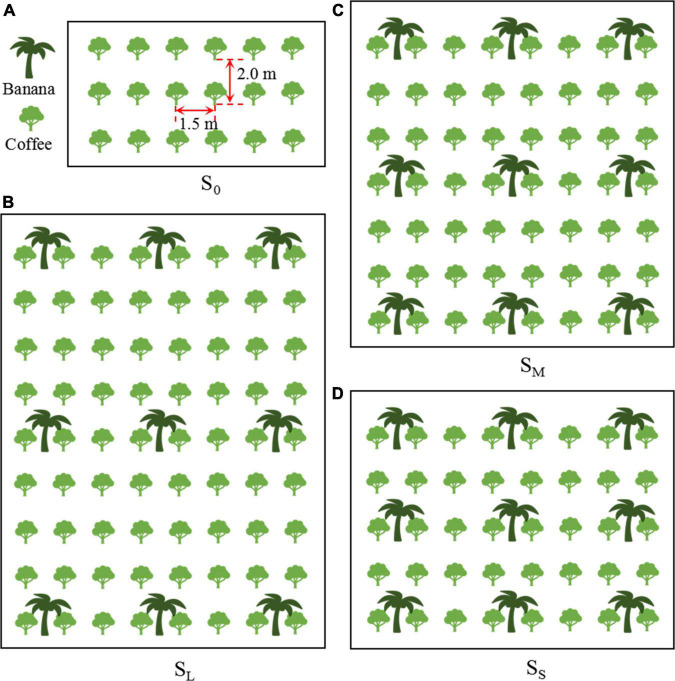
Experimental design in different shade cultivation modes. S_0_ **(A)**, S_*L*_ **(B)**, S_*M*_ **(C)**, and S_*S*_ **(D)** are no shade cultivation, light shade cultivation, moderate shade cultivation, and severe shade cultivation, respectively.

The four shade cultivation modes were no shade cultivation (S_0_: monoculture coffee; i.e., natural light intensity), light shade cultivation (S_*L*_: intercropping four lines of coffee with one line of banana, plant spacing of 4.5 m, and row spacing of 8.0 m of banana plant, i.e., 278 plants ha^–1^), moderate shade cultivation (S_*M*_: intercropping three lines of coffee with one line of banana, plant spacing of 4.5 m, and row spacing of 6.0 m of banana plant, i.e., 370 plants ha^–1^), and severe shade cultivation (S_*S*_: intercropping two lines of coffee with one line of banana, plant spacing of 4.5 m, and row spacing of 4.0 m of banana plant, i.e., 556 plants ha^–1^). When banana plants were planted to provide shade, the width of the experimental area was 10.5 m (8 coffee plants and 3 banana plants). The areas of S_0_, S_*L*_, S_*M*_, and S_*S*_ were 7.5 × 4 = 30 m^2^ (18 coffee plants and 0 banana plants), 10.5 × 16 = 168 m^2^ (72 coffee plants and 9 banana plants), 10.5 × 12 = 126 m^2^ (56 coffee plants and 9 banana plants), and 10.5 × 8 = 84 m^2^ (40 coffee plants and 9 banana plants), respectively, and the total area was 4,896 m^2^. The experimental design in different shade cultivation modes is shown in Figure 3. The purpose of the banana plant without irrigation treatment was to control the irrigation uniformity under the same shade cultivation mode. During the experiment, the banana plants did not show obvious symptoms of insufficient water and fertilizer. According to the local recommended rate of fertilizer, 500 g plant^–1^ of compound fertilizer (N:P_2_O_5_:K_2_O = 15:15:15) was applied on 12 May 2016, 26 August 2016, 6 May 2017, and 24 August 2017, respectively. The fertilizer was uniformly applied to a 20-cm-deep circular groove, 40-cm away from the coffee tree trunk. Manual weeding was performed monthly. Pests and insects were controlled in early May. Diflubenzuron was sprayed manually, and the coffee trees were not pruned. Weeding and pest control are consistent with the routine management of local coffee farmers.

### Plant Sampling and Measurements

Soil bulk density and field capacity were determined by the cutting ring method. Soil pH was measured using a standard pH meter with a soil/water ratio of 1/2.5. Soil organic matter, available N, available P, and available K content were determined using the K_2_Cr_2_O_7_-external heating method, KCl extraction, molybdenum antimony anti-colorimetry, and CH_3_COONH_4_ extraction, respectively.

Growth indexes of coffee were measured at the beginning of the experiment (11 March 2016) and at harvest (16 January 2018), respectively. The difference between the two measured values was analyzed as an increment. Height, crown width, and shoot length of coffee were measured by a mm ruler. The stem diameter of coffee was measured by a digital Vernier caliper.

Photosynthetic characteristics of functional leaves in the same direction were determined every 2 h using a portable photosynthesis system (LI-6400XT, United States). The measurements were carried out from 10:00 to 16:00 on the 2nd to 6th day after irrigation in a typical irrigation period in the fruit expansion phase (23–27 December 2016) and flowering and fruit-setting phase (6–10 May 2017) of coffee, respectively. The measurement indicators include the leaf net photosynthetic rate (*Pn*, μmol CO_2_ m^–2^ s^–1^), transpiration rate (*Tr*, mmol H_2_O m^–2^ s^–1^), intercellular CO_2_ concentration (*Ci*, μmol H_2_O mol^–1^), stomatal conductance (*Gs*, mmol CO_2_ m^–2^ s^–1^), and photosynthetically active radiation (*PAR*, μmol m^–2^ s^–1^). The instantaneous water-use efficiency of the leaf (*LWUE*, μmol mmol^–1^) is the ratio of the *Pn* to *Tr*, and the radiation use efficiency (*RUE*, μmol mmol^–1^) is the ratio of the *Pn* to *PAR*.

Bright-red and purple-red mature beans were harvested in batches at the end of 2016 and 2017, respectively. Submerging after molting, they were washed, kneaded, and shelled after static fermentation, and then degummed. The bean yield was determined after they had been dried in natural sunlight, and their quality was determined after crushing, shelling, grinding, and sieving (Liu et al., 2018). The quality indicators were total sugar, protein, fat, caffeine, chlorogenic acid, crude fiber, and water extract content. Among these indicators, the content of total sugar, protein, fat, crude fiber, and water extract was determined using anthrone colorimetry, Kjeldahl, Soxhlet extraction, acid-base digestion, and boiling water reflux extraction, respectively, whereas the content of caffeine and chlorogenic acid was determined using high-performance liquid chromatography (Liu et al., 2016, 2018; Negi et al., 2021; Sun et al., 2022). Ripe bananas were harvested in batches, and the economic benefits of the bananas were calculated after the yield was determined at the end of 2017.

### Economic Analysis

Based on the local coffee bean purchase prices of USD 2.85 kg^–1^ in 2016 and USD 2.22 kg^–1^ in 2017, the 2-year average price of USD 2.54 kg^–1^ was used in the economic benefit evaluation. The banana purchase price was USD 0.22 kg^–1^. The annual fertilizer cost was USD 1,215.85 ha^–1^ (USD 0.36 kg^–1^). The costs of irrigation water and banana seedlings were USD 0.079 m^–3^ and USD 0.56 plant^–1^, respectively, and the labor costs of banana planting, banana harvesting, and coffee bean harvesting were calculated as USD 2.38 plant^–1^, USD 0.79 plant^–1^, and USD 19.03 day^–1^, respectively. According to the experience of coffee farmers over many years, other annual labor costs were determined to account for 20% of the total investment (including weeding, fertilization, spraying, pruning, degumming, and baking the beans).

The net income (*NI*) is the total income (*TR*) minus the total input (*TC*):


(1) NI=TR-TC

The total income is the total value of the harvested dried coffee beans and bananas:


(2) $$TR=Y_{c}\times P_{c}+Y_{b}\times P_{b}$$

where *Y*_*c*_ and *Y*_*b*_ are the yields of *coffee* and bananas, respectively, and *P*_*c*_ and *P*_*b*_ are the unit prices of *coffee* beans and bananas, respectively.

The return (*R*) can be used to determine the optimal use of the system inputs (Amiri et al., 2019), which is the ratio of the net income to total input (Chen et al., 2017):


(3) R=(NI/TC)×100%

### Basic Principles and Procedure of Principal Component Analysis

(1) The data matrix ***R*** of the evaluation objects and evaluation indicators was established: there were 3 × 4 (three irrigation levels and four shade cultivation modes) evaluation objects and seven (content of total sugar, protein, fat, caffeine, chlorogenic acid, crude fiber, and water extract) evaluation indicators:


(4) $$R={\left(r_{ij}\right)}_{m\times n}$$

where *r*_*ij*_ is the original data of the *j*th evaluation index in the *i*th evaluation sample, with *m* = 12 and *n* = 7.

(2) The reciprocal transformation from the low-quality index to the high-quality index obtained a new data matrix *R’* = (*r_*ij*_’*)_*m* × *n*_. In this study, caffeine and crude fiber were low-quality indexes.

(3) *R’* was standardized to obtain the new data matrix *R”*:


(5) $$R^{\prime \prime }={\left(r_{ij}^{\prime \prime }\right)}_{m\times n}$$

(4) The number of dimensions of matrix *R”* was reduced: (a) The “original analysis result” in the descriptor was selected as well as the “coefficient” and “KMO and Bartlett spherical test” in the relevance matrix. (b) The “principal component” method was selected in the extraction term, “correlation matrix” in analyses, “non-rotating factor solution” and “gravel map” in outputs, and “basic eigenvalue” in extracts. The eigenvalue was set to a number greater than 1, and the maximum number of iterations was set to 25. (c) The “none” method was selected in the rotating term and “load diagram” in outputs. (d) The score item and selection item were set to the default.

(5) Result interpretation: (a) Whether the matrix *R”* was suitable for principal component analysis was judged using the correlation matrix and spherical testing. (b) Principal components were extracted by using common factor variance, interpretation total variance, and the gravel map. (c) The component matrix *A* and component graph were analyzed:


(6) $$A={\left(a_{jl}\right)}_{n\times t}$$

where *a*_*jl*_ is the component matrix of the *l*th interpretation information in the *j*th evaluation index, *n* = 7, and *t* is the number of extracted principal components.

(6) The eigenvector matrix *Q* was calculated:


(7) $$Q={\left(q_{jl}\right)}_{n\times t}$$

(8) $$q_{jl}=a_{jl}/\lambda_{t}^{0.5}$$

whereλ_*t*_is the eigenvalue corresponding to the extracted principal component.

(7) The principal component score *Y* was calculated:


(9) $$Y=R^{\prime \prime }\cdot Q$$

(8) The comprehensive evaluation index *f* was calculated and ranked, the higher the *f*, the better the rating evaluation:


(10) $$f=\sum \left(Y\cdot \lambda_{t}/\sum \lambda_{t}\right)$$

### Basic Principles and Procedure of Cluster Analysis

(1) Selective cluster analysis is generally performed by using the systematic cluster method for small sample data (hierarchical clusters) and quick cluster method for large sample data (K-means clusters). The systematic cluster method was used in this study.

(2) The data matrix *R* was established as in Eq. (4).

(3) Clustering matrix *R*: Analysis-Classification-Systematic cluster. (a) “Merge process table” was selected in the statistics option. (b) “Inter-group association” was selected in the cluster method and “square Euclidean distance” for the metric interval. (c) The “scheme scope” was chosen among the cluster members; the minimum and maximum numbers of clusters were set to 2 and 5, respectively.

### Statistical Analysis

Data statistical analysis was performed using Microsoft Excel 2013 software, data plotting was performed using ArcGIS 10.6 and Origin 2018 software, whereas correlation analysis, principal component analysis, cluster analysis, and variance analysis were performed using IBM SPSS (v.21.0, SPSS Inc., 2013). The treatment means were compared for any significant differences using Duncan’s multiple-range tests at the *p* = 0.05 level.

## Results

### Photosynthetic Characteristics

The effects of irrigation level on the transpiration rate (*Tr*), intercellular CO_2_ concentration (*Ci*), stomatal conductance (*Gs*) and radiation use efficiency (*RUE*) of coffee leaf were significant (*p* < 0.05), the effects of shade cultivation mode on the net photosynthetic rate (*Pn*), *Tr*, *Ci*, *Gs*, and *RUE* of coffee leaf were significant (*p* < 0.05), but the interaction effects between the irrigation level and shade cultivation mode on photosynthetic characteristics of coffee were non-significant (*p* > 0.05; Table 1).

**TABLE 1:** Effects of deficit irrigation on photosynthetic characteristics of *C. arabica* leaf under different shade cultivation modes.

Stages	Irrigation level	Shade cultivation mode	*Pn*	*Tr*	*Ci*	*Gs*	*LWUE*	*RUE*
			(μmol m^–2^ s^–1^)	(mmol m^–2^ s^–1^)	(μmol mol^–1^)	(mmol m^–2^ s^–1^)	(μmol mmol^–1^)	(μmol mmol^–1^)
Fruit expansion phase	FI	S_0_	2.57 ± 0.48ab	2.22 ± 0.33ab	321.38 ± 6.36ef	28.21 ± 0.56e	1.25 ± 0.16ns	8.52 ± 1.11g
		S_*L*_	3.04 ± 0.68ab	2.60 ± 0.35a	321.68 ± 6.35ef	31.91 ± 0.63b	1.27 ± 0.21ns	16.36 ± 2.72ef
		S_*M*_	3.23 ± 0.67a	2.63 ± 0.35a	315.57 ± 6.24f	33.93 ± 0.67a	1.32 ± 0.20ns	22.83 ± 3.36bc
		S_*S*_	2.76 ± 0.48ab	2.32 ± 0.32ab	314.08 ± 6.19f	32.25 ± 0.64b	1.29 ± 0.16ns	27.68 ± 3.40a
	DI_*L*_	S_0_	2.36 ± 0.42ab	1.96 ± 0.29b	333.39 ± 6.60bcd	24.81 ± 0.49g	1.20 ± 0.05ns	7.49 ± 0.90g
		S_*L*_	2.80 ± 0.60ab	2.32 ± 0.31ab	336.85 ± 6.65abc	28.33 ± 0.56de	1.21 ± 0.10ns	14.30 ± 2.24f
		S_*M*_	2.95 ± 0.58ab	2.34 ± 0.31ab	328.92 ± 6.50cde	29.99 ± 0.59c	1.26 ± 0.09ns	19.91 ± 2.77cde
		S_*S*_	2.50 ± 0.41ab	2.03 ± 0.28b	323.37 ± 6.37def	28.08 ± 0.55e	1.24 ± 0.04ns	24.25 ± 2.77ab
	DI_*S*_	S_0_	2.28 ± 0.42b	1.93 ± 0.29b	342.30 ± 6.65ab	24.27 ± 0.49g	1.17 ± 0.05ns	6.97 ± 0.88g
		S_*L*_	2.70 ± 0.59ab	2.27 ± 0.31ab	344.33 ± 6.67a	27.68 ± 0.56ef	1.19 ± 0.10ns	13.12 ± 2.16f
		S_*M*_	2.83 ± 0.57ab	2.28 ± 0.30ab	334.49 ± 6.49abc	29.14 ± 0.59d	1.24 ± 0.09ns	18.03 ± 2.64de
		S_*S*_	2.38 ± 0.40ab	1.96 ± 0.27b	327.00 ± 6.32cde	27.03 ± 0.55f	1.21 ± 0.05ns	21.28 ± 2.61bcd
Flowering and fruit-setting phase	FI	S_0_	2.73 ± 0.46ns	2.30 ± 0.28abc	326.27 ± 7.99cde	29.75 ± 0.69de	1.18 ± 0.06ns	8.71 ± 1.19f
		S_*L*_	3.10 ± 0.64ns	2.55 ± 0.30abc	326.96 ± 8.51cde	32.37 ± 0.81c	1.21 ± 0.11ns	15.25 ± 2.60de
		S_*M*_	3.33 ± 0.55ns	2.71 ± 0.29a	321.61 ± 9.54de	35.05 ± 0.98a	1.25 ± 0.08ns	22.01 ± 3.13b
		S_*S*_	3.01 ± 0.48ns	2.48 ± 0.28abc	319.20 ± 8.89e	33.30 ± 1.01b	1.21 ± 0.06ns	27.78 ± 3.63a
	DI_*L*_	S_0_	2.61 ± 0.44ns	2.18 ± 0.27bc	339.75 ± 6.00abc	27.14 ± 0.37g	1.19 ± 0.05ns	8.16 ± 1.11f
		S_*L*_	2.99 ± 0.62ns	2.44 ± 0.29abc	341.70 ± 7.72ab	29.76 ± 0.45de	1.22 ± 0.11ns	13.50 ± 2.27e
		S_*M*_	3.22 ± 0.54ns	2.61 ± 0.28ab	332.46 ± 9.76bcde	32.40 ± 0.63c	1.25 ± 0.08ns	18.52 ± 2.52bcd
		S_*S*_	2.93 ± 0.47ns	2.38 ± 0.27abc	326.47 ± 9.01cde	30.70 ± 0.63d	1.23 ± 0.06ns	22.14 ± 2.74b
	DI_*S*_	S_0_	2.47 ± 0.42ns	2.09 ± 0.27c	344.86 ± 7.82ab	25.51 ± 0.06h	1.18 ± 0.05ns	7.65 ± 1.04f
		S_*L*_	2.81 ± 0.55ns	2.34 ± 0.28abc	350.54 ± 9.13a	28.06 ± 0.15f	1.20 ± 0.10ns	12.53 ± 1.95e
		S_*M*_	3.07 ± 0.50ns	2.52 ± 0.28abc	339.49 ± 11.09abc	30.62 ± 0.34d	1.24 ± 0.07ns	17.36 ± 2.27cd
		S_*S*_	2.77 ± 0.44ns	2.30 ± 0.26abc	333.81 ± 9.36bcd	28.96 ± 0.35e	1.20 ± 0.06ns	20.41 ± 2.51bc
ANOVA results								
Fruit expansion phase	I		Ns	**	**	**	ns	**
	S		*	*	**	**	ns	**
	I × S		Ns	ns	ns	ns	ns	ns
Flowering and fruit-setting phase	I		Ns	*	**	**	ns	**
	S		*	**	**	**	ns	**
	I × S		Ns	ns	ns	ns	ns	ns

*FI, DI_L_ and DI_S_ are full irrigation, light deficit irrigation and severe deficit irrigation, respectively.*

*S_0_, S_L_, S_M_ and S_S_ are no shade cultivation, light shade cultivation, moderate shade cultivation and severe shade cultivation, respectively.*

*I is irrigation level, S is shade cultivation mode.*

*Different small letters in the same column indicated significant difference at 0.05 level.*

* means significant difference (p < 0.05), ** means significant difference (p < 0.01), while ns means no significant difference (p > 0.05).

In the flowering and fruit-setting stage, compared with full irrigation (FI), light deficit irrigation (DI_*L*_) decreased the *Gs* and *RUE* by 8.0% and 15.5%, respectively; severe deficit irrigation (DI_*S*_) decreased the *Pn*, *Tr*, *Gs*, and *RUE* by 8.6, 7.9, 13.3, and 21.4%, respectively, but increased the *C*_*i*_ by 5.8%. Compared with monoculture coffee (S_0_), light shade cultivation (S_*L*_) increased the *Pn*, *Tr*, *Gs*, and *RUE* by 14.0, 11.6, 9.5, and 68.4%, respectively, moderate shade cultivation (S_*M*_) increased the *Pn*, *Tr*, *Gs*, instantaneous water-use efficiency of the leaf (*LWUE*) and *RUE* by 23.2, 19.3, 19.0, 5.4, and 136.1, respectively, severe shade cultivation (S_*S*_) increased the *Pn*, *Tr*, *Gs*, and *RUE* by 11.4, 9.0, 12.8, and 186.8%, respectively.

In the fruit expansion stage, compared with the FI treatment, the DI_*L*_ treatment decreased the *Pn*, *Tr*, *Gs*, and *RUE* by 8.5, 11.4, 12.0, and 12.5%, respectively; the DI_*S*_ treatment decreased the *Pn*, *Tr*, *Gs*, *LWUE*, and *RUE* by 12.2, 13.4, 14.4, 6.2, and 21.2%, respectively, but increased the *C*_*i*_ by 5.9%. Compared with the S_0_ treatment, the S_*L*_ treatment increased the *Pn*, *Tr*, *Gs*, and *RUE* by 18.5, 17.7, 13.8, and 90.6%, respectively, the S_*M*_ treatment increased the *Pn*, *Tr*, *Gs*, *LWUE*, and *RUE* by 25.0, 18.7, 20.4, 5.5, and 164.6%, respectively, the S_*S*_ treatment increased the *Pn*, *Gs*, and *RUE* by 5.8, 13.0, and 218.7%, respectively.

Taking the photosynthetically active radiation (*PAR*) as the independent variable, the *RUE* as the dependent variable, the best fitting model was established through curve estimation regression analysis (Table 2). The results showed that the *RUE* and *PAR* had a significant negative exponential relationship when the irrigation level was the same, and the *RUE* and *PAR* conformed to the logistic curve relationship when the shade cultivation was the same.

**TABLE 2:** Regression model of radiation use of *C. arabica* leaf under different shade cultivation modes and deficit irrigation.

Treatments	The regression model of *PAR* and *RUE* in fruit expansion stage	R^2^	*F*-test (Sig.)	*t*-test (Sig.)		The regression model of *PAR* and *RUE* in flowering and fruit-setting stage	R^2^	*F*-test (Sig.)	*t*-test (Sig.)	
				α	*B*				α	*B*
FI	*RUE* = 51.520e^–0.006^*^PAR^*	0.9978	0.001	0.001	0.002	*RUE* = 51.397e^–0.0057^*^PAR^*	0.9977	0.001	0.001	0.002
DI_*L*_	*RUE* = 44.896e^–0.006^*^PAR^*	0.9980	0.001	0.001	0.001	*RUE* = 46.331e^–0.0055^*^PAR^*	0.9974	0.001	0.001	0.002
DI_*S*_	*RUE* = 40.169e^–0.005^*^PAR^*	0.9965	0.002	0.002	0.002	*RUE* = 43.356e^–0.0054^*^PAR^*	0.9956	0.002	0.002	0.003
S_0_	*RUE* = 0.0102^–1^ × 1.0082^–^*^PAR^*	0.9800	0.090	0.001	0.223	*RUE* = 0.0013^–1^ × 1.0144^–^*^PAR^*	0.9758	0.099	0.001	0.394
S_*L*_	*RUE* = 0.0075^–1^ × 1.0113^–^*^PAR^*	0.9816	0.087	0.001	0.188	*RUE* = 0.0104^–1^ × 1.0090^–^*^PAR^*	0.9468	0.148	0.001	0.276
S_*M*_	*RUE* = 0.0053^–1^ × 1.0152^–^*^PAR^*	0.9705	0.110	0.002	0.237	*RUE* = 0.0120^–1^ × 1.0088^–^*^PAR^*	0.9779	0.095	0.001	0.138
S_*S*_	*RUE* = 0.0043^–1^ × 1.0216^–^*^PAR^*	0.9504	0.143	0.003	0.305	*RUE* = 0.0114^–1^ × 1.0106^–^*^PAR^*	0.9784	0.094	0.001	0.124

*FI, DI_L_ and DI_S_ are full irrigation, light deficit irrigation and severe deficit irrigation, respectively.*

*S_0_, S_L_, S_M_ and S_S_ are no shade cultivation, light shade cultivation, moderate shade cultivation and severe shade cultivation, respectively.*

*RUE is radiation use efficiency (6.01-31.16 μmol mmol^–1^); PAR is photosynthetically active radiation (96.01-344.78 μmol m^–2^ s^–1^).*

*α means coefficient of PAR in the fitted model; B means coefficient of constant term in the fitted model.*

### Growth

**FIGURE 4:**
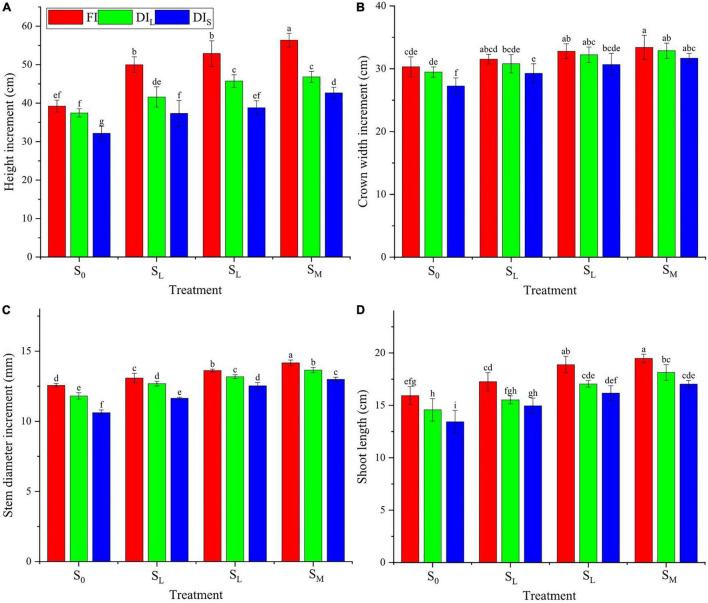
Effects of deficit irrigation on growth (height increment, **A**; crown width increment, **B**; stem diameter increment, **C**; shoot length, **D**) of *C. arabica* under different shade cultivation modes.

The effects of the irrigation level and shade cultivation mode on the increment of height, crown width, and stem diameter and shoot length of coffee were significant (*p* < 0.05), and their interaction on height increment and stem diameter increment of coffee was significant (*p* < 0.05; Figure 4). Compared with the FI treatment, the DI_*L*_ treatment decreased the height increment and shoot length by 13.5 and 8.8%, respectively; the DI_*S*_ treatment decreased the height increment, crown width increment, stem diameter increment, and shoot length by 23.9, 7.1, 10.6, and 13.9%, respectively. Compared with the S_0_ treatment, other shade cultivation modes increased the height increment, crown width increment, stem diameter increment, and shoot length by 18.5–34.0%, 5.3–12.6%, 6.9–16.6%, and 8.6–24.4%, respectively. Compared with the FIS_0_ treatment, the DI_*S*_S_0_ treatment decreased the height increment by 18.0%, the DI_*L*_S_0_, DI_*S*_S_*L*,_ and DI_*S*_S_*M*_ treatments had no significant effects, and the other treatments increased it by 8.7–43.6%; the FIS_*M*_, FIS_*S*,_ and DI_*L*_S_*S*_ treatments increased stem diameter increment by 8.4, 12.7, and 8.6%, respectively, the DI_*L*_S_0_, DI_*S*_S_0,_ and DI_*S*_S_*L*_ treatments decreased it by 6.0, 15.5, and 7.4%, respectively, and the other treatments had no significant effects.

### Bean Yield

**FIGURE 5:**
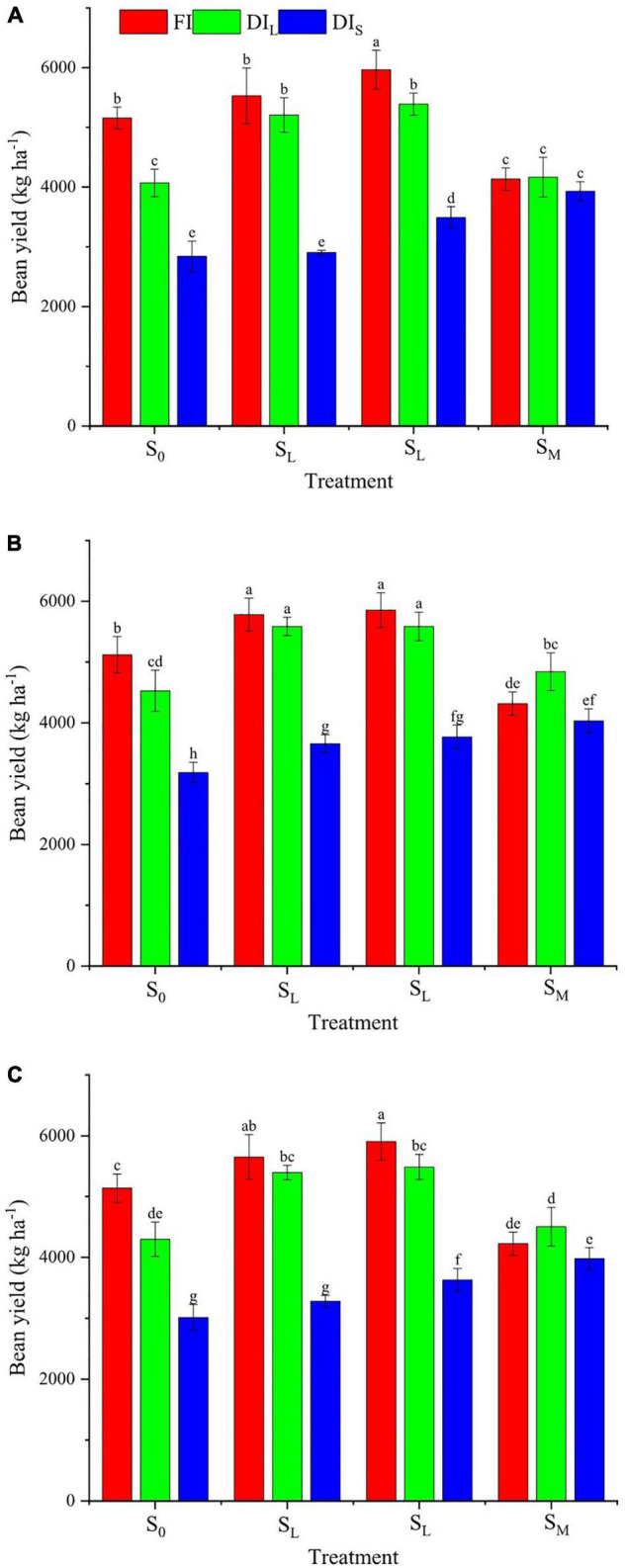
Effects of deficit irrigation on bean yield of *C. arabica* under different shade cultivation modes in 2016 **(A)**, 2017 **(B)**, and 2-year average **(C)**.

The effects of irrigation level, shade cultivation mode, and their interaction on the bean yield of coffee were significant (*p* < 0.05; Figure 5). The yield increased with an increase in the degree of shade when water was severely deficient, whereas, under full irrigation and light deficit irrigation, it first increased and then decreased with the increasing shade degree. In 2016, the yield was between 2,840 and 5,966 kg ha^–1^. Compared with the FIS_0_ treatment, the FIS_*M*_ treatment increased the yield by 15.7%. The FIS_*L*_, DI_*L*_S_*L*_, and DI_*L*_S_*M*_ treatments non-significantly increased the yield, whereas the other treatments decreased the yield by 19.2–44.9%. In 2017, the yield was between 3,184.2 and 5,854.3 kg ha^–1^. Compared with the FIS_0_ treatment, the DI_*L*_S_*S*_ treatment non-significantly decreased the yield; the FIS_*L*_, FIS_*M*_, DI_*L*_S_*L*_, and DI_*L*_S_*M*_ treatments increased the yield by 12.9, 14.3, 9.1, and 9.1%, respectively, whereas the other treatments decreased the yield by 11.6–37.8%; the FIS_*S*_ treatment decreased the yield by 15.7%. The 2-year average yield was between 3,012 and 5,910 kg ha^–1^. Compared with the FIS_0_ treatment, the DI_*L*_S_*L*_ and DI_*L*_S_*M*_ treatments non-significantly increased the 2-year average yield; the FIS_*L*_ and FIS_*M*_ treatments increased the 2-year average yield by 10 and 15%, respectively, but the other treatments decreased the 2-year average yield by 12.3–41.4%, among them, the FIS_*S*_ and DI_*S*_S_0_ treatments decreased the 2-year average yield by 17.8 and 41.4%, respectively.

### Nutritional Quality

The effects of the irrigation level on the total sugar content of coffee were non-significant (*p* > 0.05), but the effects of irrigation level on protein, fat, caffeine, chlorogenic acid, crude fiber, and water extract content were significant (*p* < 0.05) (Table 3). The effects of shade cultivation mode on all nutritional quality indicators of coffee were also significant (*p* < 0.05). The interaction effects between irrigation level and shade cultivation mode on fat and crude fiber content of coffee were non-significant (*p* > 0.05); however, the interaction effects on the other nutritional quality indicators were significant (*p* < 0.05). Compared with the FIS_0_ treatment, the FIS_*M*_, DI_*L*_S_*L*,_ and DI_*S*_S_*S*_ treatments increased the total sugar content by 6.1, 4.3, and 6.1%, respectively; the DI_*S*_S_0_ treatment decreased it by 3.8%, and the other treatments had no significant effects. The DI_*L*_S_0_ treatment decreased the protein content by 5.3%; the FIS_*L*_, DI_*S*_S_0,_ and DI_*S*_S_*L*_ treatments had non-significant effects; and other treatments increased it by 3.3–19.9%, among them, the FIS_*M*_ and FIS_*S*_ treatments increased the protein content by 10.8 and 19.9%, respectively. The FIS_*L*_ and DI_*L*_S_*L*_ treatments decreased the caffeine content by 13.7 and 8.8%, respectively, but the other treatments increased it by 3.9–21.6%, among them, the FIS_*M*_ and FIS_*S*_ treatments increased the caffeine content by 7.8 and 3.9%, respectively. The DI_*S*_S_0_ treatment decreased the chlorogenic acid content by 7.8%, whereas the DI_*L*_S_0_ treatment had a non-significant effect; the other treatments increased it by 7.2–43.1%, among them, the FIS_*M*_ and FIS_*S*_ treatments increased the chlorogenic acid content by 40.6 and 43.1%, respectively. The DI_*S*_S_0_ treatment decreased the water extract content by 7.6%; the DI_*L*_S_0_ and DI_*S*_S_*L*_ treatments had non-significant effects, and the other treatments increased it by 3.4–11.9%, among them, the FIS_*M*_ and FIS_*S*_ treatments increased the water extract content by 10.0 and 11.9%, respectively.

**TABLE 3:** Effects of deficit irrigation on nutritional quality of *C. arabica* under different shade cultivation modes.

Irrigation level	Shade cultivation mode	Total sugar [g (100g) ^–1^]	Protein [g (100g) ^–1^]	Fat [g (100g) ^–1^]	Caffeine [g (100g) ^–1^]	Chlorogenic acid (g kg^–1^)	Crude fiber [g (100g) ^–1^]	Water extracts [g (100g) ^–1^]
FI	S_0_	9.33 ± 0.19cde	16.50 ± 0.22fg	12.10 ± 0.37c	1.02 ± 0.01g	13.85 ± 0.05g	17.10 ± 0.32de	39.20 ± 0.45g
	S_*L*_	9.55 ± 0.13bc	16.78 ± 0.13ef	12.38 ± 0.41abc	0.89 ± 0.02i	15.71 ± 0.04e	16.78 ± 0.24e	40.95 ± 0.42f
	S_*M*_	9.90 ± 0.14a	18.28 ± 0.15cd	12.65 ± 0.19ab	1.10 ± 0.01d	19.47 ± 0.48b	16.13 ± 0.30f	43.10 ± 0.33bc
	S_*S*_	9.43 ± 0.15cd	19.78 ± 0.21a	12.78 ± 0.25a	1.06 ± 0.01f	19.82 ± 0.42a	14.38 ± 0.36h	43.88 ± 0.51a
DI_*L*_	S_0_	9.58 ± 0.15bc	15.63 ± 0.17h	11.40 ± 0.36de	1.08 ± 0.02de	13.59 ± 0.05g	17.93 ± 0.32bc	38.93 ± 0.44g
	S_*L*_	9.53 ± 0.15bc	17.05 ± 0.24e	11.88 ± 0.29cd	0.93 ± 0.03h	15.41 ± 0.05e	17.33 ± 0.33de	40.53 ± 0.26f
	S_*M*_	9.10 ± 0.18ef	18.90 ± 0.29b	12.10 ± 0.18c	1.10 ± 0.01d	15.47 ± 0.08e	17.00 ± 0.28e	42.70 ± 0.22cd
	S_*S*_	9.73 ± 0.17ab	19.05 ± 0.24b	12.23 ± 0.19bc	1.16 ± 0.01c	17.65 ± 0.30d	14.93 ± 0.31g	43.33 ± 0.46ab
DI_*S*_	S_0_	8.98 ± 0.15f	16.33 ± 0.22g	11.30 ± 0.42e	1.24 ± 0.01a	12.77 ± 0.06h	19.28 ± 0.48a	36.23 ± 0.31h
	S_*L*_	9.18 ± 0.13def	16.38 ± 0.21g	11.43 ± 0.22de	1.21 ± 0.01b	14.84 ± 0.07f	18.18 ± 0.51b	38.63 ± 0.29g
	S_*M*_	9.58 ± 0.17bc	18.50 ± 0.29c	12.08 ± 0.34c	1.07 ± 0.01ef	15.63 ± 0.05e	17.58 ± 0.46cd	41.65 ± 0.26e
	S_*S*_	9.90 ± 0.32a	17.98 ± 0.25d	12.13 ± 0.43c	1.09 ± 0.01de	18.33 ± 0.03c	15.73 ± 0.26f	42.35 ± 0.53d
ANOVA results								
I		ns	**	**	**	**	**	**
S		**	**	**	**	**	**	**
I × S		**	**	ns	**	**	ns	**

*FI, DI_L_ and DI_S_ are full irrigation, light deficit irrigation and severe deficit irrigation, respectively.*

*S_0_, S_L_, S_M_ and S_S_ are no shade cultivation, light shade cultivation, moderate shade cultivation and severe shade cultivation, respectively.*

*I is irrigation level, S is shade cultivation mode.*

*Different small letters in the same column indicated significant difference at 0.05 level.*

** means significant difference (p < 0.01), while ns means no significant difference (p > 0.05).

The results of the Pearson correlation analysis of the nutritional quality indexes of coffee (Table 4) revealed significant positive correlations between the total sugar content, chlorogenic acid content, and water extract content. In addition, significant positive correlations were discovered between protein and fat content; chlorogenic acid and water extract content; fat, chlorogenic acid, and water extract content; and chlorogenic acid and water extract content. Crude fiber content was negatively correlated with total sugar, protein, fat, chlorogenic acid, and water extract content. Therefore, it can be concluded that benefit evaluation of nutritional quality cannot be conducted comprehensively and scientifically only through the analysis of the correlations between indicators.

**TABLE 4:** Correlation coefficient matrix of not baking bean nutritional quality of *C. arabica*.

Nutritional quality index	Total sugar	Protein	Fat	Caffeine	Chlorogenic acid	Crude fiber	Water extracts
Total sugar	1						
Protein	0.277	1					
Fat	0.514	0.751**	1				
Caffeine	−0.321	0.032	−0.441	1			
Chlorogenic acid	0.650*	0.785**	0.834**	−0.102	1		
Crude fiber	−0.587*	−0.771**	−0.843**	0.235	−0.881**	1	
Water extracts	0.593*	0.881**	0.859**	0.199	0.878**	−0.887**	1

** and * represents p < 0.01 and p < 0.05, respectively.

### Principal Component Analysis of Nutritional Quality Indexes

The Kaiser–Meyer–Olkin (KMO) and Bartlett’s test revealed that the KMO statistic was 0.74 > 0.5, indicating that the sample size was sufficient. The result of Bartlett’s sphericity test was *p* < 0.05, which demonstrated that the hypothesis of sphericity was rejected and indicated that the seven quality indicators were related. The findings showed that the nutritional quality indexes of coffee are suitable for principal component analysis.

**FIGURE 6:**
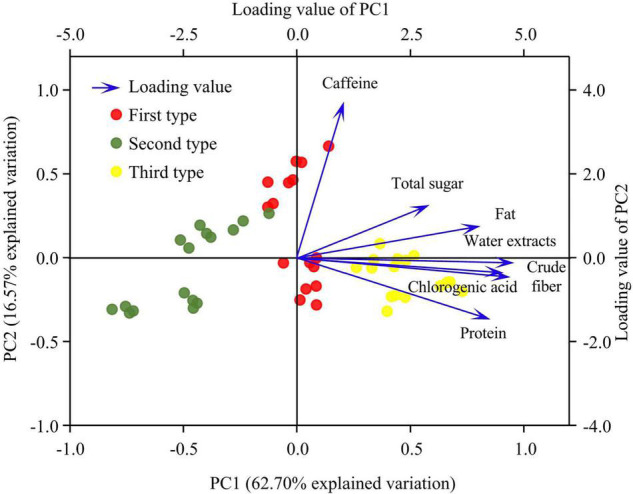
Results of the principal component analysis (PCA).

The characteristic root of the first principal component was 4.74, and this component explained 62.7% of the total variance, the size of the principal component was mainly determined by protein, fat, chlorogenic acid, crude fiber, water extract, and total sugar content, among which total sugar content had the weakest influence (Figure 6). The characteristic root of the second principal component was 1.2, and this component explained 16.5% of the total variance, the size of the principal component was mainly determined by caffeine content (Figure 6). The first and second principal components explained 85% of the total variance.

The eigenvector was computed using the component matrix and extracted the principal component eigenvalue:


(11) Q1=4.738A-0.51

(12) Q2=1.179A-0.52

where *A*_1_ and *A*_2_ are the first and second component matrixes, respectively, and *Q*_1_ and *Q*_2_ are the first and second eigenvectors, respectively.

Combining the standardized vector of the nutritional quality index (Table 5) and the eigenvector, the expression of the principal component was determined, and the comprehensive score was calculated:


(13) $$y_{\text{1}}=0.\text{3}0\text{3}T_{S}+0.\text{39}0P_{RO}+0.\text{423}F_{AT}+0.\text{1}0\text{3}C_{AF}+0.\text{435}C_{A}+0.\text{426}C_{F}+0.\text{443}W_{E}$$

(14) $$y_{\text{2}}=0.\text{328}T_{S}-0.\text{367}P_{RO}+0.\text{144}F_{AT}+0.\text{846}C_{AF}-0.\text{113}C_{A}-0.0\text{83}C_{F}-0.0\text{46}W_{E}$$

(15) $$f=\left(\text{4}.\text{738}/\text{5}.\text{917}\right)\times y_{\text{1}}+\left(\text{1}.\text{179}/\text{5}.\text{917}\right)\times y_{\text{2}}$$

**TABLE 5:** Principal component analysis of the nutritional quality index of *C. arabica*.

Irrigation Level	Shade cultivation mode	Standardized vector of nutritional quality index							*y*_1_	*y*_2_	*f*	Ranking
		total sugar	protein	fat	caffeine	chlorogenic acid	crude fiber	water extracts				
FI	S_0_	−0.523	−0.829	0.137	0.518	−0.959	−0.240	−0.755	−1.223	0.754	−0.829	9
	S_*L*_	0.240	−0.621	0.722	2.104	−0.147	−0.017	−0.002	0.282	2.209	0.666	5
	S_*M*_	1.427	0.516	1.306	−0.268	1.493	0.456	0.923	2.410	−0.009	1.928	2
	S_*S*_	−0.184	1.653	1.571	0.105	1.647	1.943	1.257	3.365	−0.756	2.544	1
DI_*L*_	S_0_	0.325	−1.492	−1.350	−0.120	−1.072	−0.771	−0.873	−2.248	0.583	−1.684	10
	S_*L*_	0.155	−0.412	−0.341	1.487	−0.275	−0.390	−0.185	−0.471	1.483	−0.082	8
	S_*M*_	−1.285	0.990	0.137	−0.273	−0.252	−0.173	0.751	0.176	−0.987	−0.056	7
	S_*S*_	0.833	1.104	0.403	−0.776	0.701	1.438	1.020	2.142	−0.975	1.521	3
DI_*S*_	S_0_	−1.709	−0.962	−1.563	−1.377	−1.427	−1.540	−2.035	−3.874	−1.215	−3.344	12
	S_*L*_	−1.031	−0.924	−1.297	−1.178	−0.526	−0.922	−1.002	−2.408	−1.001	−2.128	11
	S_*M*_	0.325	0.687	0.084	−0.038	−0.181	−0.552	0.299	0.216	−0.113	0.151	6
	S_*S*_	1.427	0.289	0.190	−0.184	0.998	0.767	0.601	1.633	0.029	1.313	4
Eigenvector	***Q*_1_**	0.303	0.390	0.423	0.103	0.435	0.426	0.443				
	***Q*_2_**	0.328	−0.367	0.144	0.846	−0.113	−0.083	−0.046				

*FI, DI_L_ and DI_S_ are full irrigation, light deficit irrigation and severe deficit irrigation, respectively.*

*S_0_, S_L_, S_M_ and S_S_ are no shade cultivation, light shade cultivation, moderate shade cultivation and severe shade cultivation, respectively.*

*y_1_ and y_2_ are first and second principal component score, respectively, f is integrated score.*

***Q_1_** and **Q_2_** are first and second eigenvector, respectively.*

where *y*_1_ and *y*_2_ are the first and second principal component scores, respectively, *f* is the integrated score, and *T*_*S*_, *P*_*RO*_, *F*_*AT*_, *C*_*AF*_, *C*_*A*_, *C*_*F*_, and *W*_*E*_ are the standardized vector of total sugar, protein, fat, caffeine, chlorogenic acid, crude fiber, and water extract content, respectively.

The treatments, ordered by their comprehensive evaluation score from high to low, were FIS_*S*_, FIS_*M*_, DI_*L*_S_*S*_, DI_*S*_S_*S*_, FIS_*L*_, DI_*S*_S_*M*_, DI_*L*_S_*M*_, DI_*L*_S_*L*_, FIS_0_, DI_*L*_S_0_, DI_*S*_S_*L*_, and DI_*S*_S_0_. Thus, the highest comprehensive benefit was obtained using the FIS_*S*_ treatment, followed by the FIS_*M*_ treatment, and the lowest benefit was obtained using the DI_*S*_S_0_ treatment.

### Cluster Analysis of Nutritional Quality Indexes

**FIGURE 7:**
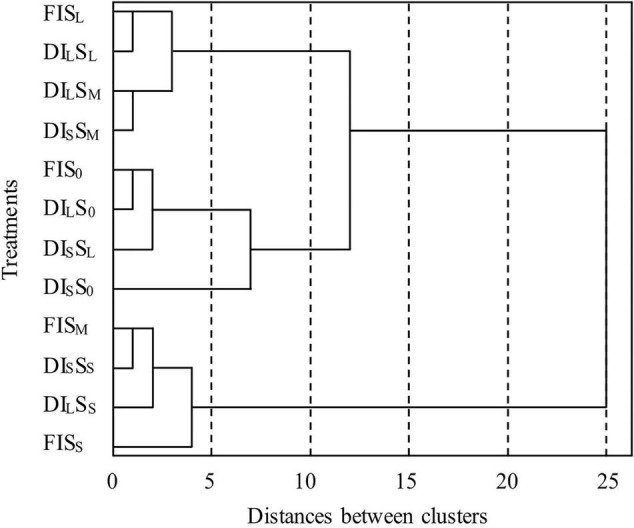
Cluster dendrogram of seven indicators of the nutritional quality index of *C. arabica.* FI, DI_*L*,_ and DI_*S*_ are full irrigation, light deficit irrigation, and severe deficit irrigation, respectively. S_0_, S_*L*_, S_*M*_, and S_*S*_ are no shade cultivation, light shade cultivation, moderate shade cultivation, and severe shade cultivation, respectively.

The cluster analysis of the nutritional quality indexes of coffee, performed using the hierarchical clustering method (Figure 7), indicated that the twelve treatments could be divided into three types when the distance between the classes was 10: the first type, FIS_*L*_, DI_*L*_S_*L*_, DI_*L*_S_*M*_, and DI_*S*_S_*M*_ treatments; the second type, FIS_0_, DI_*L*_S_0_, DI_*S*_S_*L*_, and DI_*S*_S_0_ treatments; and the third type, FIS_*M*_, DI_*S*_S_*S*_, DI_*L*_S_*S*,_ and FIS_*S*_ treatments. The third type had the largest average irrigation amount and highest average shade degree, whereas the second type had the lowest average irrigation amount and lowest average shade degree. The average irrigation amount and average shading degree of the first type were between those of the second and third types. When the distance between the classes was 3, the second type could be divided into two subtypes: the first subtype, FIS_0_, DI_*L*_S_0_, and DI_*S*_S_*L*_ treatments; the second subtype was DI_*S*_S_0_ treatment. Additionally, the third type could be divided into two subtypes the first subtype, FIS_*M*_, DI_*S*_S_*S*_, and DI_*L*_S_*S*_ treatments; the second subtype was FIS_*S*_ treatment (this subtype had the highest score and comprehensive quality in principal component analysis).

### Economic Benefit

In this study, the input of banana seedling planting was added in 2016 compared with 2017, and the economic income of banana was added in 2017 compared with 2016 (Table 6). Compared with the FIS_0_ treatment, the FIS_*L*_, FIS_*M*_, DI_*L*_S_*L*_, and DI_*L*_S_*M*_ treatments increased the 2-year average total revenue by 22.6, 29.5, 17.0, and 20.6%, respectively; the increases obtained using the FIS_*S*_ and DI_*L*_S_*S*_ treatments were non-significant, and the other treatments decreased the revenue by 7.8–41.4%. The FIS_*L*_, FIS_*M*_, DI_*L*_S_*L*_, and DI_*L*_S_*M*_ treatments increased the 2-year average net income by 22.1, 28.5, 16.5, and 18.9%, respectively; the DI_*L*_S_*S*_ treatment had a non-significant effect, but the other treatments decreased the income by 8.7–47.4%, among them, the FIS_*M*_ treatment decreased the 2-year average net income by 8.7%. The FIS_*L*_, FIS_*M*_, and DI_*L*_S_*L*_ treatments non-significantly decreased the 2-year average return, and the DI_*L*_S_*M*_ and DI_*L*_S_*S*_ treatments decreased it by 9.5 and 5.5%, respectively. The other treatments decreased the return by 31.2–34.4%, among them, the FIS_*S*_ treatment decreased the 2-year average return by 31.7%.

**TABLE 6:** Effects of deficit irrigation on the economic benefit of *C. arabica* under different shade cultivation modes.

Irrigation	Shade	Input of irrigation		Input of	Input of harvest coffee		Total input		Income of coffee beans		Income of	Two-year average	Two-year average	Two-year
level	cultivation mode	(USD ha^–1^)		banana (USD ha^–1^)	beans (USD ha^–1^)		(USD ha^–1^)		(USD ha^–1^)		banana (USD ha^–1^)	total revenue (USD ha^–1^)	net income (USD ha^–1^)	average return (%)
		2016	2017	2016	2016	2017	2016	2017	2016	2017	2017			
FI	S_0_	390	359	–	982	974	3,235	3,187	13,097 ± 464b	13,007 ± 755b	–	13,052 ± 603c	9,841 ± 603c	306.5 ± 18.8a
	S_*L*_	390	359	816	1,052	1,100	4,343	3,619	14,040 ± 1,187b	14,684 ± 685a	3,269 ± 198de	15,997 ± 888b	12,016 ± 888ab	301.8 ± 22.3ab
	S_*M*_	390	359	1,086	1,136	1,113	4,785	3,727	15,153 ± 828a	14,870 ± 722a	3,780 ± 252c	16,902 ± 757a	12,646 ± 757a	297.1 ± 17.8ab
	S_*S*_	390	359	1,631	788	822	5,032	3,548	10,503 ± 475c	10,966 ± 488de	5,082 ± 498a	13,276 ± 709c	8,986 ± 709c	209.5 ± 16.5c
DI_*L*_	S_0_	293	269	–	775	862	2,854	2,934	10,336 ± 587c	11,501 ± 858cd	–	10,918 ± 717e	8,024 ± 717d	277.3 ± 24.8b
	S_*L*_	293	269	816	992	1,064	4,145	3,462	13,229 ± 737b	14,192 ± 381a	3,109 ± 142e	15,265 ± 341b	11,462 ± 341b	301.4 ± 9.0ab
	S_*M*_	293	269	1,086	1,026	1,064	4,525	3,553	13,693 ± 472b	14,190 ± 597a	3,606 ± 209cd	15,744 ± 575b	11,705 ± 575b	289.8 ± 14.2ab
	S_*S*_	293	269	1,631	794	921	4,917	3,559	10,583 ± 842c	12,302 ± 787bc	4,700 ± 268b	13,792 ± 745c	9,554 ± 745c	225.4 ± 17.6c
DI_*S*_	S_0_	195	180	–	541	605	2,439	2,501	7,213 ± 656e	8,088 ± 429h	–	7,651 ± 541g	5,181 ± 541f	209.7 ± 21.9c
	S_*L*_	195	180	816	552	697	3,473	2,891	7,374 ± 101e	9,291 ± 377g	2,961 ± 324e	9,813 ± 362f	6,631 ± 362e	208.4 ± 11.4c
	S_*M*_	195	180	1,086	664	717	3,951	3,008	8,863 ± 466d	9,573 ± 495fg	3,201 ± 248e	10,819 ± 566e	7,339 ± 566de	210.9 ± 16.3c
	S_*S*_	195	180	1,631	748	767	4,738	3,254	9,980 ± 415c	10,240 ± 504ef	3,839 ± 418c	12,029 ± 337d	8,033 ± 337d	201.0 ± 8.4c

*FI, DI_L_ and DI_S_ are full irrigation, light deficit irrigation and severe deficit irrigation, respectively.*

*S_0_, S_L_, S_M_ and S_S_ are no shade cultivation, light shade cultivation, moderate shade cultivation and severe shade cultivation, respectively.*

*Different small letters in the same column indicated significant difference at 0.05 level.*

## Discussion

Water is an important raw material that affects the photosynthesis of crops. Intensification of water deficit will cause the disintegration of crop cell membranes, and lead to the degradation of chlorophyll and enhanced photoinhibition (Hao et al., 2017). Light is the most important energy source for the photosynthesis of crops. Shade cultivation will inevitably cause changes in leaf photosynthesis, transpiration, and the source–sink relationship, which will affect the absorption and utilization of water by crops (Ren et al., 2016; Liu et al., 2017). This study demonstrated that the *Pn*, *Tr*, *Gs*, and *LWUE* of coffee leaves increased first and then decreased with the increase of shade degree, and *RUE* increased with the increase of shade degree. The main reason is that the shade cultivation affects the temperature and humidity of the canopy, changes the vapor pressure deficit, and affects the stomata opening of the leaves, which in turn changes the photosynthetic characteristics of coffee leaves. The *Pn*, *Tr*, *Gs*, *LWUE*, and *RUE* of coffee leaves decreased with the increase of water deficit, and *Ci* increased with the increase of water deficit. It may be due to the lack of soil moisture that causes the leaf stomata to close and the CO_2_ supply is limited. It may be also due to the increased CO_2_ diffusion resistance of the mesophyll cells and the decreased activity of photosynthetic enzymes (Hu et al., 2017; Yi et al., 2017). With the increase of shade degree and decrease of water deficit, the *Pn*, *Tr*, *Gs*, *LWUE*, and *RUE* of coffee leaf increased in varying degrees, while *Ci* decreased in varying degrees. It shows that under the condition of sufficient soil moisture, moderate shade cultivation can make the coffee obtain higher water and radiation use efficiency of leaf, which is like the results of Liu et al. (2017). The increase in shade degree and the decrease in water deficit will change the canopy microenvironment, intercept light radiation, decrease the temperature, increase the humidity of the canopy, then adjust the stomata opening and transpiration water consumption, and promote photosynthesis and obtain higher water-radiation use efficiency.

Under the same irrigation level, the radiation use efficiency (*RUE*) and photosynthetically active radiation (*PAR*) of the leaf showed a significant negative exponential relationship, indicating the *RUE* decreased rapidly and then slowly with the increase of the *PAR*. When the *PAR* increased to a certain extent, the *RUE* is maintained at the same level, which is consistent with the results of previous studies (Liu et al., 2017). Under the same shade cultivation mode, the *RUE* and the *PAR* conformed to the logistic curve, indicating the *RUE* decreased slowly and then rapidly decreased with the increase of the *PAR*, and finally decreased slowly. The reason may be that the photosynthesis of crops was greatly restricted when the water deficit was excessive. The decrease in the net photosynthetic rate was the main reason for the low radiation use efficiency.

When water deficit occurs during crop growth, their physiological metabolism will change, which leads to a change in growth conditions. Proper increase of soil moisture can promote root development, expand the contact area between root and soil, which is beneficial to increase nutrient absorption and speed up nutrient transport. Most scholars believed that shading can greatly promote the vegetative growth of crops. Moderate shading can promote the growth of crops, increase the height and stem diameter of the crop, develop the root system, flourish branches and leaves, and increase the root-shoot ratio (Liu et al., 2017). This study found that the height increment, crown width increment, stem diameter increments, and shoot length of coffee all decrease with the increase of water deficit, and increase with the increase of shade degree, which indicated that DI inhibits the vegetative growth of coffee. A certain degree of shade cultivation conditions can enhance the physiological activity, and increase relative growth rate, which is consistent with the results of previous studies (Bosselmann et al., 2009; Sakai et al., 2015).

Different water and light environments have differing effects of induction and regulation on the synthesis and accumulation of secondary metabolites in the organs, tissues, and cells of crops, ultimately affecting the crop yield and quality (Cramer et al., 2011). This study discovered that the bean yield of coffee increased with an increase in the degree of shade when water was severely lacking. This may have been because shade cultivation decreases the ambient temperature, reducing transpiration water consumption and soil surface water evaporation (Padovan et al., 2018); it also mitigates the pressure of water stress, so the normal fruit setting of coffee is unaffected or less affected (Ramalho et al., 2018). When irrigation is full or only lightly deficient, the bean yield of coffee first increased and then decreased with an increase of the shade degree. Moreover, the maximum bean yield in 2016 was that occurring under moderate shade cultivation (S_*M*_). In 2017, the bean yields were similar between light shade cultivation (S_*L*_) and moderate shade cultivation (S_*M*_), but the bean yield of light shade cultivation (S_*L*_) was slightly higher than that of moderate shade cultivation (S_*M*_). This can be explained by the branches and leaves of the banana plants getting wider over time, so a light shade degree in 2017 was equivalent to a moderate shade degree in 2016. Additionally, this finding indicates that shade cultivation is a crucial factor determining the higher yield of coffee when soil moisture is suitable, which is related to the growth habit of coffee in cool, moist, and shady environments (Liu et al., 2018). The average bean yield in 2017 was slightly higher than that in 2016, which was related to the larger cumulative rainfall and strong shade effect of the banana plants in 2017. The bean yield when shade cultivation was severe (S_*S*_) was significantly lower, which was due to the less flower bud differentiation and lesser fruit of coffee because of excessive shade, resulting in low bean yield; this finding may also have been caused by the water and fertilizer competition between banana plants and coffee (Meylan et al., 2017).

Crop quality is often affected by environmental factors, and reasonable water and light allocation can achieve simultaneous optimization of yield and quality (Brooker et al., 2015; Zhou et al., 2020). This study discovered that irrigation level, shade cultivation mode, and the interaction of these two factors had significant effects on most of the nutritional quality indicators of the coffee beans. This may have been because irrigation in a shade environment relieves soil drought stress, improves the photosynthetic characteristics of the plant, promotes the accumulation and conversion of photosynthetic products, and delays the maturity of coffee berries (Martins et al., 2019). Moreover, water affects the physiological metabolism of plants and the absorption, transportation, and transformation of inorganic and organic substances, thus influencing the nutrient content of coffee beans (Ali et al., 2020). The chlorogenic acid content of coffee beans increased with an increase in the irrigation amount and shade degree. The reason may be that the primary productivity of the plants was greatly inhibited under a severe soil water deficit and the raw materials used in the synthesis of secondary products were scarce (Li et al., 2017; Liu et al., 2018). However, increasing the amount of irrigation and shade had a dilution effect on the accumulation of chlorogenic acid (de Lazzari Almeida et al., 2017). In addition, the more shade the environment provides, the more effectively evaporation and transpiration operate between trees. Moreover, the higher the environmental humidity is, the more the chlorogenic acid in the coffee beans is diluted. Different shade environments inevitably lead to different source–storage relationships, resulting in varying hydrolysis degrees of proteins, starches, fats, and so on in the coffee beans. This study discovered that the levels of protein, fat, and water extract in coffee beans increased with an increase in the shade degree, indicating that a certain shade environment improves the ability of the source organs of coffee to supply assimilation, enhancing the volume of coffee beans. The source organ is strong in supply, whereas the overall acceptance of the storage organ is weak, so the coffee beans were highly enriched, and the occurrence of chalkiness was reduced (Dwivedi et al., 2013), improving the nutritional quality of the coffee beans (Rueda et al., 2017).

In practical production, multiple variables are employed to conduct comprehensive evaluations and address the problem systematically. However, intricate correlations exist between these variables, such as multiple collinearities, which can cause large errors in the analysis of real problems. Principal component analysis, cluster analysis, fuzzy comprehensive evaluation, TOPSIS method, the analytic hierarchy process, and the gray correlation method are used by domestic and foreign scholars to comprehensively evaluate crop quality (Wang and Xing, 2017; Wang et al., 2019, 2020; Liu et al., 2020; Peng and Deng, 2020; Xing et al., 2022). Different evaluation schemes have different calculation methods. In this study, cluster analysis indicated that the 12 treatments could be divided into five types by using hierarchical clustering; the FIS_*S*_ and DI_*S*_S_0_ treatments were divided into their type. However, the principal component analysis revealed that the FIS_*S*_ treatment had the highest comprehensive quality, whereas the DI_*S*_S_0_ treatment had the lowest. The FIS_*M*_, DI_*S*_S_*S*_, and DI_*L*_S_*S*_ treatments were treated as one type in cluster analysis, and they ranked second, third, and fourth in principal component analysis, respectively; their comprehensive quality was high. This was different from the results of Liu et al. (2018), who reported that the highest comprehensive benefit was that obtained using DI75Sh30 treatment (75% of full irrigation amount, 30% of shading natural light intensity), followed by FISh30 treatment (full irrigation amount, 30% of shading natural light intensity), with the lowest obtained using the DI50Sh0 treatment (50% of full irrigation amount, natural light intensity). The reason for this discrepancy may be that the shade environment for coffee was provided by the banana plant in this study, and the shade was dynamic; this was different from the shade environment provided by black shading nets in Liu et al. (2018). Additionally, the environmental ventilation, temperature and humidity, and absorption and utilization of soil water and fertilizer were substantially different between the two studies. Moreover, the discrepancy may also be related to the experimental irrigation amount, the difference in precipitation, and the difference in nutritional quality indicators analyzed. Some relevant information is unknown because only the nutritional quality of the coffee beans produced in the second year of banana shade cultivation was determined in this study; whether the nutritional quality of the coffee beans under different shade cultivation durations varies should be systematically studied. In this experimental area, coffee beans sold by coffee farmers have not been graded, and this study can only refer to the purchase price of local coffee beans when analyzing economic benefits. This shortcoming of the study would be considered in our follow-up studies.

Crop yield and quality are the preferred indicators when determining the economic benefits of coffee cultivation, and improving the yield and quality is the basis of achieving high yield and efficiency (Perdoná and Soratto, 2015b; Liu et al., 2018; Yang et al., 2021). The water supply and light environment are crucial factors affecting the yield and quality of coffee, and reasonable coordination of water–light relationship can achieve low input and high output. This study demonstrated that the effects of irrigation level and shade cultivation mode on economic benefit were significant. Under different water and light conditions, the economic benefit of coffee varied, and the 2-year average net income of coffee was between USD 52,181 ha^–1^ and USD 12,646 ha^–1^. The FIS_*M*_ treatment obtained the highest 2-year average net income. The highest 2-year average return was not obtained using the FIS_*M*_ treatment, because the total investment was higher due to the higher investment in shade plant (banana) cultivation and management. However, the increase in net income was smaller than the increase in total investment, resulting in a lower return. In this study, the income from coffee was calculated according to the purchase price of local coffee beans, but the benefit obtained from different-quality coffee beans would differ, which requires investigation in a follow-up study.

Although this study showed that the FIS_*S*_ treatment resulted in the highest comprehensive quality, the 2-year average yield from this treatment was only 4,226.19 kg ha^–1^, the 2-year average net income was only USD 8,986 ha^–1^, and the 2-year average return was only 209.5%. Compared with the FIS_0_ treatment, the 2-year average yield, net income, and return were 17.8, 8.7, and 31.7% lower, respectively. The FIS_*M*_ treatment ranked second in terms of comprehensive quality but obtained the highest 2-year average yield of 5,910.14 kg ha^–1^, highest 2,-year average net income of USD 12,646 ha^–1^, and a 2-year average return of 297.1%, compared with the FIS_0_ treatment, the yield and net income were 15.0 and 28.5% higher, respectively, and the 2-year average return was non-significantly lower (3.1% lower). In summary, FI significantly improved the nutritional quality of coffee under intercropping with two lines of coffee for each line of banana plants (FIS_*S*_) and significantly increased the dry bean yield and economic benefits of coffee under intercropping with three lines of coffee for one line of banana plants (FIS_*M*_). The results of this study provide a theoretical basis for agricultural water supply and shade cultivation management of coffee in the dry–hot region of Yunnan, southwest China.

## Conclusion

The effects of the irrigation level, shade cultivation mode, and their interaction on the yield and most nutritional quality indicators of coffee were significant (*p* < 0.05). Compared with the FIS_0_ treatment, the FIS_*M*_ treatment increased the total sugar, protein, chlorogenic acid, and water extract content and 2-year average yield, total revenue, and net income by 6.1, 10.8, 40.6, 10.0, 15.0, 29.5, and 28.5%, respectively. When the FIS_*S*_ treatment was used, the contents of protein, chlorogenic acid, and water extract were increased by 19.9, 43.1, and 11.9%, respectively, the 2-year average bean yield and net income were decreased by 17.8 and 8.7%, respectively, and the 2-year average total revenue was increased non-significantly.

To improve nutritional quality, full irrigation under severe shade cultivation (FIS_*S*_) should be used because it is the best combination of coffee irrigation treatment and banana shade cultivation. To improve yield and increase economic output, full irrigation under moderate shade cultivation (FIS_*M*_) is the optimal combination of coffee irrigation treatment and banana shade cultivation. To summarize, the results of this study provide guidance regarding irrigation and light management of coffee in the subtropical monsoon climate region in southwest China.

## Data Availability Statement

The original contributions presented in the study are included in the article/supplementary material, further inquiries can be directed to the corresponding author/s.

## Author Contributions

XL and XW conceptualized the study. KH and XL designed the study. LF and LL performed data checks. KH and XW wrote the original draft preparation. FJ, YL, QY, and YS edited and revised the original manuscript. All authors have read and agreed to the published version of the manuscript.

## Conflict of Interest

YS was employed by the company Dehong HeiRou Coffee Co., Ltd. The remaining authors declare that the research was conducted in the absence of any commercial or financial relationships that could be construed as a potential conflict of interest.

## Publisher’s Note

All claims expressed in this article are solely those of the authors and do not necessarily represent those of their affiliated organizations, or those of the publisher, the editors and the reviewers. Any product that may be evaluated in this article, or claim that may be made by its manufacturer, is not guaranteed or endorsed by the publisher.
